# Proximity-Based Emergency Response Communities for Patients With Allergies Who Are at Risk of Anaphylaxis: Clustering Analysis and Scenario-Based Survey Study

**DOI:** 10.2196/13414

**Published:** 2019-08-22

**Authors:** Michal Gaziel Yablowitz, Sabine Dölle, David G Schwartz, Margitta Worm

**Affiliations:** 1 The Graduate School of Business Administration Bar-Ilan University Ramat Gan Israel; 2 Allergy-Centre-Charite Department of Dermatology and Allergy Charite-Universitatsmedizin Berlin Berlin Germany

**Keywords:** consumer health informatics, anaphylaxis, emergency responders, social networking, telemedicine

## Abstract

**Background:**

Anaphylaxis is a potentially fatal allergic reaction. However, many patients at risk of anaphylaxis who should permanently carry a life-saving epinephrine auto injector (EAI) do not carry one at the moment of allergen exposure. The proximity-based emergency response communities (ERC) strategy suggests speeding EAI delivery by alerting patient-peers carrying EAI to respond and give their EAI to a nearby patient in need.

**Objectives:**

This study had two objectives: (1) to analyze 10,000 anaphylactic events from the European Anaphylaxis Registry (EAR) by elicitor and location in order to determine typical anaphylactic scenarios and (2) to identify patients’ behavioral and spatial factors influencing their response to ERC emergency requests through a scenario-based survey.

**Methods:**

Data were collected and analyzed in two phases: (1) clustering 10,000 EAR records by elicitor and incident location and (2) conducting a two-center scenario-based survey of adults and parents of minors with severe allergy who were prescribed EAI, in Israel and Germany. Each group received a four-part survey that examined the effect of two behavioral constructs—shared identity and diffusion of responsibility—and two spatial factors—emergency time and emergency location—in addition to sociodemographic data. We performed descriptive, linear correlation, analysis of variance, and *t* tests to identify patients’ decision factors in responding to ERC alerts.

**Results:**

A total of 53.1% of EAR cases were triggered by food at patients’ home, and 46.9% of them were triggered by venom at parks. Further, 126 Israeli and 121 German participants completed the survey and met the inclusion criteria. Of the Israeli participants, 80% were parents of minor patients with a risk of anaphylaxis due to food allergy; their mean age was 32 years, and 67% were women. In addition, 20% were adult patients with a mean age of 21 years, and 48% were female. Among the German patients, 121 were adults, with an average age of 47 years, and 63% were women. In addition, 21% were allergic to food, 75% were allergic to venom, and 2% had drug allergies. The overall willingness to respond to ERC events was high. Shared identity and the willingness to respond were positively correlated (r=0.51, *P*<.001) in the parent group. Parents had a stronger sense of shared identity than adult patients (t_243_= –9.077, *P*<.001). The bystander effect decreased the willingness of all patients, except the parent group, to respond *(F*_1,269_=28.27, *P*<.001). An interaction between location and time of emergency (*F*_1,473_=77.304, *P*<.001) revealed lower levels of willingness to respond in strange locations during nighttime.

**Conclusions:**

An ERC allergy app has the potential to improve outcomes in case of anaphylactic events, but this is dependent on patient-peers’ willingness to respond. Through a two-stage process, our study identified the behavioral and spatial factors that could influence the willingness to respond, providing a basis for future research of proximity-based mental health communities.

## Introduction

### Background

Anaphylaxis is a serious, potentially fatal, systemic allergic reaction with a rapid onset. Symptoms of anaphylaxis range from skin and mucosal tissue changes such as urticaria and angioedema to life-threatening respiratory and cardiovascular conditions [1]. Anaphylaxis affects the lives of 49 million individuals in the United States alone and international guidelines consider it a medical emergency that requires rapid intervention [2-4]. To prevent potentially fatal anaphylaxis, patients with severe allergy are advised to permanently carry one or more doses of epinephrine in auto injector form (EAI). With the onset of symptoms, the immediate intramuscular injection of epinephrine from the personal EAI is used as lifesaving treatment until the arrival of emergency medical services (EMS) for continuing care. However, a significant proportion of adult patients and caregivers of minor patients with EAI prescription do not always carry their EAI and thereby expose themselves to life threats, which may not be quickly answered by EMS [5-7].

**Figure 1:**
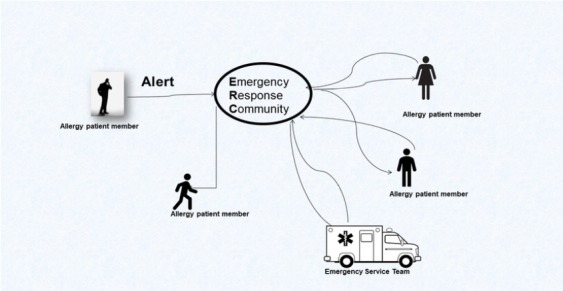
Proximity-based emergency response community during an anaphylactic event.

Research suggests that nearby patient-peers who maintain long-term EAI prescriptions can potentially deliver their personal EAI to the patient in need through a proximity-based emergency response communities (ERC) app [8,9]. As described in Figure 1, ERC apps dispatch nearby registered patients with allergy to help a patient in immediate need of an EAI, in certain configurations following the approval of EMS.

Based on the proximity of patient-peers, the ERC intervention is dependent, in part, on factors such as population density, prescription density, and EAI availability [10] as well as the willingness of patients to join ERCs [11]. The intervention is also dependent on the willingness of patients to respond to a stranger’s request on short notice and in unfamiliar circumstances.

Prior studies that examined the efficacy of proximity-based mobile health (mHealth) interventions in improving emergency outcomes point to the insufficient participation rates as a major unsolved challenge [12-15]. Studies discussing helping behavior between strangers suggest the dual effect of diffused responsibility and shared identity as a potential explanation [16-18].

Indeed, a central factor that can impact the willingness to respond and help among a crowd of strangers is diffused responsibility, also known as the bystander effect [18-20]. According to the bystander effect, when a person has noticed an emergency event, the mere knowledge of other witnessing bystanders can decrease willingness to respond [21]. In an ERC scenario, the bystander effect may manifest when an emergency request is transmitted to a patient-peer, given that they know there are many more users of the app. This knowledge can decrease the willingness to respond at the single user level, leading to no community response. Nevertheless, the shared identity between patient-peers may serve to counteract the bystander effect, leading to mutual aid [16,22-26]. Indeed, studies about group identity among peer chronic patients show their strong willingness to help each other during crises [26].

Spatial and temporal factors such as the time of emergency event can limit this helping motivation. Studies show that the willingness to help a distressed stranger varies during daytime and nighttime. Research found that women’s fear of attackers in public areas during the night decreases their willingness to respond [27]. On the other hand, men are less likely to respond at night due to a lower motivation for volunteering activity [28]. Hence, the time of emergency occurrence can affect the willingness to respond. Researchers pointed to the level of familiarity with event location that may affect the willingness to travel from the responder’s location to the victim’s spot, as it demands an increase in the cognitive effort needed for traveling in a potentially unfamiliar environment [29]. In ERC events, the willingness of ERC members to respond can decrease if the incident location is unfamiliar and demands a concentrated effort.

Furthermore, the willingness to respond might be limited by the high cost of EAI in some communities [30]. To examine the influence of these behavioral and spatial decision factors on the response decision to ERC alerts among adult patients and parents of minor patients with EAI prescription, we conducted a scenario-based survey across two centers: (1) The Yahel Israeli Foundation for Food Allergy in Israel (Yahel), where 73% of the members are parents of minor patients with food allergy and 27% are adult patients, and (2) the outpatient clinic at the Department of Dermatology, Venereology and Allergology, Charité - Universitätsmedizin Berlin in Germany (Charité), where more than 90% of the patients are adults with allergies. Studies point to high levels of volunteering activity in Israel and Germany, suggesting that their willingness to help a distressed patient-peer may not be affected despite cultural differences [31,32]. In addition, both centers are characterized by the low cost of EAI due to a national health insurance program allowing elimination of the EAI cost from the list of behavioral barriers to respond to ERC alerts in these communities.

Finally, data analysis of the European Anaphylaxis Registry (EAR) of anaphylaxis events [33,34], collected by 90 tertiary allergy centers in 10 European countries, will allow us to develop scenarios of typical acute anaphylactic events that will reflect the emergency events of an ERC allergy app.

The improved knowledge of the behavioral and spatial decision factors would be useful in designing interventions that support patients with allergy to respond to and potentially improve emergency outcomes.

### Objectives

The main goal of this study is to identify behavioral and spatial factors influencing patients at risk of an acute anaphylactic event in responding to emergency events and providing their personal EAI to a nearby patient-peer in need through a scenario-based survey. To determine typical acute anaphylactic scenarios, we analyzed the EAR dataset with 10,000 records of anaphylactic events.

## Methods

### Recruitment

Between May 2017 and June 2018, we conducted a scenario-based survey that was distributed in The Yahel Israeli Foundation for Food Allergy in Israel, whose members are parents of minor patients with food allergy and adult patients, and the outpatient clinic at the Department of Dermatology, Venereology and Allergology, Charité - Universitätsmedizin Berlin in Germany, which mainly treats adult patients.

Inclusion criteria for both centers were as follows: current diagnosis of allergy, considered to be at continuing risk for anaphylactic reaction, and current EAI prescription.

Exclusion criteria for both centers were as follows: at low risk for anaphylactic reaction as per an assessment and no current EAI prescription.

A total of 126 Israeli participants, members of the official Facebook page of Yahel, were recruited consecutively through a post. The post invited group members at risk of anaphylactic events and having an EAI prescription to participate in a survey. Members who wished to participate were directed through a link to answer an external online four-part survey.

Although recruitment through Facebook may lead to a selection bias in which the most motivated group members will respond, the distribution of a detailed four-part survey among group members with a majority of parents of minor patients with food allergies during their summer vacation months leads to a low motivation to respond [35]. To increase responsiveness, personal messages were sent to the group members every 3 days and the post was repeated periodically over a period of 2 months. Eventually, only 3% of Yahel Facebook members were recruited.

**Figure 2:**
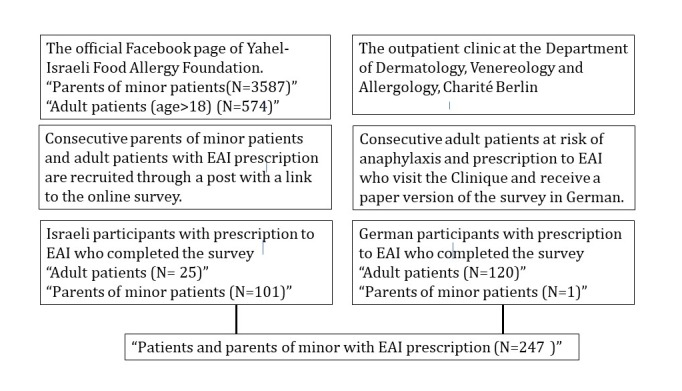
Recruitment process of study participants. EAI: epinephrine auto injector.

A total of 121 German patients at risk of an anaphylactic event and with an EAI prescription, who visited the Charité outpatient clinic, were recruited in a consecutive manner during the same period. Figure 2 described the selection process of the dual sampling methodology of Israeli and German participants.

### Questionnaires and Scenario Development

The survey was built to examine the effect of shared identity, bystander effect [19,21,36], time of emergency [27,37], and location familiarity [38,39] on willingness to respond to anaphylaxis events through an ERC app. The questionnaire contained four sections:

The first section covered the participants’ anonymous personal data and information on allergy status (Multimedia Appendix 1).

In the second section, participants were given an explanation of the ERC app for allergy patients and its potential lifesaving activity during acute anaphylactic events. Participants were assured that they will not bear any legal liability for participating in emergency events that are protected by Good Samaritan laws [22,40]. Subsequently, they were asked to project themselves as ERC members who use the app. Their initial level of shared identity was measured through an in-group identification tool [41] (Multimedia Appendix 2).

The third section of the survey presented usage scenarios [42,43] describing various anaphylaxis events when no EAI was available and help was summoned through the ERC app (Multimedia Appendix 3).

The scenarios were developed through the following two-stage process.

#### Stage 1: Categorizing European Anaphylaxis Registry Events According to Location and Triggers

The EAR records details of anaphylaxis events collected by 90 tertiary allergy centers in 10 European countries. It was created in 2007 and as of 2019, it contains nearly 14,000 records [34].

To determine scenarios that represent typical triggers and locations of acute anaphylactic events, we clustered 10,000 cases from the EAR dataset using a two-step cluster algorithm applied through SPSS [computer software] (Version: 25.0. Armonk, NY: IBM Corp). Clustering results were used to analyze the data set by two variables: (1) allergy elicitor, which included the following seven values—food, venom, latex, drugs, exercise, stress, cold and “I don’t know”—and (2) incident location, which included the following 10 values—place of work, medical practice, garden or park, urban public place, restaurant or takeaway food, friend’s home, dentist, home, school or kindergarten, public transportation, and “I don’t know.”

#### Stage 2: Developing Scenarios of Anaphylactic Events

Cluster analysis results formed the basis of the scenarios’ storylines in which location familiarity, time of emergency, and bystander effect were activated to measure their impact on willingness to respond through the levels noted in Table 1.

**Table 1:** Decision factors’ research matrix.

Bystander effect	Daytime		Nighttime	
	Unfamiliar location	Familiar location	Unfamiliar location	Familiar location
Single responder	1	2	5	7
Multiple responders	3	4	6	8

The 2 × 2 × 2 research matrix of Table 1 resulted in final eight scenarios with the following combinations:

1. Daytime, unfamiliar location, a single responder
2. Daytime, familiar location, a single responder
3. Daytime, unfamiliar location, multiple responders
4. Daytime, familiar location, multiple responders
5. Nighttime, unfamiliar location, a single responder
6. Nighttime, familiar location, a single responder
7. Nighttime, unfamiliar location, multiple responders
8. Nighttime, familiar location, multiple responders

To improve participants’ ability to project themselves into scenarios, the locations of incidents and the main character’s personal details were attuned to each study region. Following presentation of the scenario, participants were asked to rate their willingness to respond to ERC emergency requests considering the scenario circumstances. Finally, to validate our selection of scenarios’ location, we asked participants to rate their familiarity levels with each scenario location through a location familiarity questionnaire (Multimedia Appendix 4).

#### Data Collection

Each group was divided into two subgroups. Each Israeli subgroup received a different online version of a survey through a closed Facebook page of Yahel foundation. Each of these versions contained two scenarios. Each German subgroup received a similar paper version of the survey with two scenarios each, distributed at the Charité. None of the groups was made aware of the other survey version. All participants were given an explanation of the ERC app for allergy patients and its role in calling responders during anaphylactic events.

### Measures

Developed in English, Hebrew, and German, the survey was built to examine the effect of shared identity, bystander effect, time of emergency, and location familiarity on willingness to respond to anaphylactic events through an ERC app using 18 items measured on a 10-point Likert scale. All questions were piloted for comprehensibility and content validity on an independent sample of 40 patients randomly selected and not included in the study sample. Minor adjustments were made to the first draft after the pilot study. The scenarios were validated for content by a group of 25 allergy patients not included in the study sample. Their feedback indicated that the scenarios were reasonable and that the participants were able to put themselves in the hypothetical position of the ERC responding member (Multimedia Appendix 5). Participants’ anonymous personal data and information on allergy status were collected through a five-item demographic questionnaire. To measure shared identity, participants were asked to rate their level of agreement or disagreement with an eight item in-group identification tool. To measure willingness to respond, participants were asked to rate their level of agreement or disagreement through a three-item agreement or disagreement tool, accompanied by a series of survey statements on willingness to respond. Location familiarity levels were measured using a three-item geospatial familiarity questionnaire [36]. All survey instruments are presented in the Multimedia Appendices.

### Survey Statistical Analysis

We used simple descriptive statistics to analyze participants’ sociodemographic and clinical data. To assess the strength of the association between shared identity and the willingness to respond, we obtained Pearson correlations.

We performed subgroup analyses through an independent sample *t* test to determine whether the levels of shared identity and willingness to respond differed when the sample was restricted to sociodemographic subgroups. Level of familiarity with the locations of emergency scenarios was compared using independent sample *t* test on the whole-sample level.

To test for the main effects of bystander effect, time of emergency, and location familiarity, we preformed two-way and three-way analyses of variance and *t* tests on each stratified sample: the full sample, parents of minor patients with food allergy, adult patients with food allergy, and patients with venom allergy.

## Results

### Participant Characteristics

A total of 247 questionnaires were delivered to 146 adults with severe allergy having an EAI prescription and 101 parents of children with severe allergic having an EAI prescription.

The majority of the 126 Israeli participants (80%) were parents of children with the risk of an anaphylactic episode due to food allergy, who had an EAI prescription. The mean age of this group was 32 years, and 67% were women (Table 2). The second subgroup of the Israeli patients were adult anaphylaxis patients with a long-term EAI prescription; their average age was 21 years, and 48% of them were women.

In Germany, all 121 patients with EAI prescription were adults. Their average age was 47 years, and 64% were women. Only one participant was a parent of a patient. In addition, 21% suffered from food allergy, 75% had venom allergy, 2% had drug allergies, and 2% had other allergies.

### Comparing Shared Identity and Willingness to Respond According to Sociodemographic Data

Investigating the effect of shared identity on the willingness to respond for the whole sample required examination of the possibility to bind the sample regardless of the patients’ origin.

Table 3 compares the mean scores for shared identity and willingness to respond between Israeli and German adult patients with food allergy.

**Table 2:** Baseline characteristics of the study participants (N=247).

Characteristics		Israel (n=126)	Germany (n=121)
**Gender, n (%)**			
	Male	43 (35)	43 (36)
	Female	83 (65)	78 (64)
Age: mean, median		30, 31	47, 49
**Participant type, n (%)**			
	Adult patient	25 (20)	120 (99)
	Parent of minor patient	101 (80)	1 (1)
**Elicitor, n (%)**			
	Food	126 (100)	26 (21)
	Venom	—^a^	91 (75)
	Drug	—	2 (2)
	Other	—	2 (2)

^a^Not available.

**Table 3:** Scores for shared identity and willingness to respond among Israeli and German adult patients with food allergy.

Adult patients with food allergy	Shared identity		Willingness to respond	
	n (%)	Mean (SD)	n (%)	Mean (SD)
Israeli patients	25 (20)	6.85 (2.08)	25 (20)	7.15 (1.56)
German patients	26 (21)	6.05 (1.53)	24 (20)	6.93 (2.13)

Although a statistical test was not applicable due to the limited sample size of the two groups, results indicate small differences between the scores of shared identity and willingness to respond between German and Israeli adult patients with food allergy. As such, we reported unified results for the entire sample.

Assessment of the strength of association between shared identity and the willingness to respond revealed a significant moderate positive correlation between these two constructs for the full sample (r=0.31, *P*<.001), a significant strong correlation for the parents of minor patients with food allergy (r=0.505, *P*=.03), a significant small correlation for adults with food allergy (r=0.289, *P*<.001), and a smaller correlation for patients with venom allergy (r=0.189, *P*<.001). Table 4 shows subject characteristics according to their level of shared identity and willingness to respond. An independent sample *t* test across all baseline characteristics revealed significant differences in the level of shared identity and the willingness to respond between the type of participant and the allergy elicitor. These results indicate that parents of young patients have significantly stronger shared identity (t_243_= –9.077, *P*<.001) and significantly higher levels of willingness to respond than adult patients. Patients with food allergy have a significantly stronger shared identity (t_238_=8.9, *P*<.001) and significantly higher levels of willingness to respond than patients with venom allergy.

**Table 4:** Results of *t* testing for shared identity and willingness to respond according to participants’ characteristics.

Variable		Shared identity			Willingness to respond		
		n (%)	Mean (SD)	*P* value	n (%)	Mean (SD)	*P* value
**Type of participants**							
	Adult patient	143 (57)	5.28 (2.20)	<.001	143 (57)	7.7 (2.18)	<.001
	Minor patients’ parent	102 (41)	7.56 (1.51)	<.001	102 (41)	8.7 (1.53)	<.001
**Gender**							
	Male	86 (35)	6.10 (2.24)	.36	86 (35)	8.2 (0.98)	.74
	Female	159 (65)	6.30 (2.25)	.36	159 (64)	8.15 (2.01)	.74
**Elicitor**							
	Food	152 (61)	7.08 (1.92)	<.001	152 (62)	8.5 (1.69)	<.001
	Venom	89 (36)	4.78 (2.03)	<.001	89 (36)	7.5 (2.24)	<.001
	Drug	2 (0.8)	5.43 (2.09)	.15	2 (0.8)	6.12 (3.79)	.41
	Other	2 (0.8)	7.37 (1.01)	.15	2 (0.8)	8 (1.77)	.41

### Detecting Spatial and Behavioral Decision Factors Through Emergency Response Communities Scenarios

To detect the influence of spatial and behavioral decision factors on the willingness to respond, we first developed eight ERC survey scenarios according to the results of the EAR cluster analysis.

Data were clustered into two high-quality clusters.

The first cluster included 46.9% of anaphylaxis cases. In this cluster, 100% of incidents were triggered by venom; 50% of the incidents occurred in gardens and parks, 25% occurred in public places or at the place of work, and 25% of the cases did not report the location of the incident.

The second cluster included 53.1% of cases. In this cluster, 48% of incidents were triggered by food, 30% were triggered by drugs, 8% were triggered by venom, and 14% did not report the trigger. In addition, 50% of the incidents occurred at home; 15% occurred at medical practices and hospitals; 7% occurred in restaurants and hotels; and 28% were split between school, friend’s home, urban public places, gardens and parks, place of work, and missing data.

Next, we compared the response decisions of the eight survey groups across three stratified populations.

Table 5 compares the average mean levels of willingness to respond across eight scenarios and four stratified survey populations.

**Table 5:** Mean levels of willingness to respond, for each assigned scenario across survey populations.

Scenarios	Full sample		Patients with food allergy				Patients with venom allergy (all adults, no parents)	
			Parents of minor patients		Adult patients			
	n (%)	Mean (SD)	n (%)	Mean (SD)	n (%)	Mean (SD)	n (%)	Mean (SD)
NUM^a^	55 (22)	5.95 (1.46)	—^b^	—	11 (4)	6.18 (1.34)	40 (16)	6.0 (40)
NUS^c^	55 (22)	9.2 (1.22)	1 (0.4)	10 (1)	10 (4)	9.8 (0.34)	40 (16)	9.0 (40)
DUS^d^	63 (25)	8.5 (1.67)	53 (21)	8.6 (1.57)	10 (4)	8.0 (1.90)	—	—
DUM^e^	63 (25)	8.6 (1.65)	48 (19)	8.5 (1.69)	15 (6)	8.8 (1.52)	—	—
DFS^f^	61 (25)	8.6 (2.08)	1 (0.4)	5.0 (1)	13 (5)	8.8 (2.1)	48 (19)	8.6 (2.07)
DFM^g^	58 (23)	6.1 (1.85)	—	—	14 (5)	5.8 (0.69)	44 (18)	6.22 (2.09)
NFS^h^	63 (26)	9.1 (1.42)	48 (19)	9.1 (1.47)	15 (6)	9.33 (1.24)	—	—
NFM^i^	63 (26)	8.8 (1.33)	53 (21)	8.8 (1.30)	10 (4)	8.7 (1.56)	—	—

^a^NUM: Nighttime, unfamiliar location, multiple responders.

^b^Not available.

^c^NUS: Nighttime, unfamiliar location, a single responder.

^d^DUS: Daytime, unfamiliar location, a single responder.

^e^DUM: Daytime, unfamiliar location, multiple responders.

^f^DFS: Daytime, familiar location, a single responder.

^g^DFM: Daytime, familiar location, multiple responders.

^h^NFS: Nighttime, familiar location, a single responder.

^i^NFM: Nighttime, familiar location, multiple responders.

To test the impact of the bystander effect, time of emergency, and the type of location and its interactions on the willingness to respond, we performed variance analysis on four stratified samples (Table 6). As described in Table 4, significant differences in the scores of shared identity and the willingness to respond were detected between adult patients and parents of minor patients with food and venom allergy. Table 6 presents the main effects of each treatment factor for the full sample, parents of minors with food allergy, adults with food allergy, and adults with venom allergy: A significant main effect was observed for the bystander effect (*F*_1,473_=108.20, *P*<.001) for the full sample. The post hoc *t* test analysis showed that the bystander effect significantly decreased the willingness to respond to emergency alerts among allergy patients (t_479_=8.47, *P*<.001).

The bystander effect was also observed as a significant factor for adults with food allergy (*F*_1,90_=28.33, *P*<.001) and adults with venom allergy (*F*_1,168_=100.435, *P*<.001), but not for the parents of minor patients with food allergy (*P*=.48).

To test for the impact of emergency location on the willingness to respond, we first examined patients’ familiarity with scenarios’ locations. The results of a *t* test for independent samples (t_476_=–6.9, *P*<.001) validated that patients distinguished between unfamiliar and familiar locations and decided how to respond. A two-way significant interaction was revealed between emergency location and time for the full sample (*F*_1,473_=77.304, *P*<.001) and for adults with food allergy (*F*_1,90_=13.44, *P*<.001 *)*.

A post hoc independent sample *t* test for the full sample (t_234_=–3.9, *P*<.001), and patients with food allergy (t_132_=–2.110, *P*=.04) showed that the ERC night alerts received in strange locations significantly decreased participants’ willingness to respond compared to ERC alerts received in strange locations during the day.

In addition, *t* test results for the full sample (t_243_=6.43, *P*<.001) and adults with food allergy (t_141_=3.39, *P*<.001) indicate that night alerts received in familiar locations significantly increase the willingness to respond compared to the alerts received during the day in strange locations.

A significant three-way interaction was revealed between the bystander effect, location familiarity, and time of emergency for the full sample *(F*_1,473_=88.19, *P*<.001) and for adults with food allergy (F_1,90_=69.5 *, P*<.001). The result of a post hoc *t* test for the full sample (t_116_=–11.10, *P*<.001) and adults with food allergy (t_107_=12.63, *P*<.001) indicated that the knowledge of other potential responders in range will significantly decrease the patient-peer willingness to respond to ERC night alerts received in strange locations.

**Table 6:** Main effects and interactions of the three independent variables found by three-way analysis of variance test.

Treatment factors	Full sample		Patients with food allergy				Patients with venom allergy (all adults, no parents)	
			Parents of minor patients		Adult patients			
	*F* (df)	*P* value	*F* (df)	*P* value	*F* (df)	*P* value	*F* (df)	*P* value
Bystander effect	108.202 (1, 473)	<.001	0.857 (1, 198)	0.36	28.33 (1, 90)	<.001	100.435 (1, 168)	<.001
Time of day	4.513 (1, 473)	.03	6.457 (1, 198)	0.01	4.43 (1, 90)	.04	—^a^	—
Type of location	0.547 (1, 473)	.46	4.311 (1, 198)	0.04	0.024 (1, 90)	.88	—	—
Bystander effect* Time of Day	4.177 (1,473)	.04	—	—	2.88 (1,90)	.09	—	—
Time of Day*Type of location	77.3 (1,473)	<.001	1.63 (1, 198)	0.2	13.44 (1,90)	<.001	—	—
Type of location* Bystander effect	0.282 (1,473)	.60	—	—	0.412 (1, 90)	.52	—	—
Type of Location* Bystander effect* Time of day	88.91 (1,473)	<.001	—	—	69.5 (1,90)	<.001	—	—

^a^Not available.

## Discussion

### Principal Findings

This two-center study set out to identify the behavioral and spatial decision factors influencing food and venom allergy in patients and parents of minor patients to participate in the emergency response app, in which these patients can provide their personal EAI to a patient-peer in need.

The results of this study with 247 Israeli and German participants show the following: The overall score of willingness to respond to ERC alerts was high, especially among parents of minor patients with food allergy. The overall score of shared identity among patients with food allergy was high and varied across different types of participant. The association between shared identity and the willingness to respond was strong among parents of patients with allergy and moderate among the full sample. Bystander effect significantly decreased the willingness to respond in the full sample, patients with food allergy, and patients with venom allergy. It did not decrease the willingness to respond among parents with minors having food allergy. The combination of emergency time and its location significantly affected the willingness to respond among the full sample and adults with allergy. A three-way interaction between these two spatial constructs and the bystander effect significantly decreased the willingness to respond among the full sample and adult patients with allergy.

These findings lead to the following observations. First, the high scores of willingness to respond suggest that an ERC allergy app has the potential to improve outcomes in the case of anaphylactic events. Nevertheless, the significant differences in the sense of shared identity among adults with food allergy, parents of minors with food allergy, and patients with venom allergy indicate that not all patients at risk of anaphylaxis are the same and their response decisions will differ.

Second, the low score for shared identity found among the full sample of patients with venom allergy compared to the full sample of patients with food allergy is not surprising. With a strong lobbying activity for food labeling and allergen-free public spaces, patients with allergy and their families have a stronger common ground than those who are allergic to venom [44,45]. The high score of shared identity found among parents of minors with food allergy compared to adult patients with food allergy can be attributed to the former’s strong parental identity as caregivers of chronically ill children who share a common medical condition [46,47].

Third, the significant strong correlation between shared identity and the willingness to respond found among parents with food allergy along with their high score of shared identity and willingness to respond confirm the results of previous studies about the association between active bystander intervention and high sense of shared identity, especially among patient-peers [22,26,48-51].

The bystander effect, which was identified as a significant barrier to response [17,21,52] among the full sample, including adults with food allergy and patients with venom allergy, did not impact the willingness to respond among parents of minor patients with food allergy. These results show that patients will not always respond to ERC alerts, irrespective of the circumstances, and are selective in their response decision. Hence, there is a low possibility that participants’ response decisions were biased due to a social desirability effect. The use of an anonymous online instrument for half of the survey population further reduces the chance of social desirability bias [53,54].

These results also suggest that an effective ERC allergy app should combine the members’ proximity and strong collective identity. Thus, designing such proximity-based apps for local social community grouping of severe allergy patients can increase response rates to ERC alerts; in other words, being coincidentally in proximity is not as effective as feeling you belong to a community group. This conclusion matches what we know about the strong ability of peer patients’ local communities to benefit from health care delivery and improve health behavior among its members due to strong social cohesion and local networks [55,56].

Fourth, spatial decision factors decrease the response rate only when combined. The interaction between the time of emergency and its location, which was significant for the full sample and adults with food allergy showed that response to anaphylactic events would be limited in a strange location during the night. In accordance, a three-way interaction between ERC night alert, strange location, and the bystander effect will significantly decrease the response scores.

Indeed, studies reported low rates of prosocial behavior during nighttime due to fear, especially when the emergency occurs in unfamiliar locations [27,37]. Nevertheless, the results of post hoc *t* tests for the interaction between time and emergency location identified participants’ favorability to respond to night alerts over day alerts in unfamiliar locations. This further corresponds with studies showing that bystander intervention occurs when it helps the victim and does not harm the helper [57,58]. Hence, the fear of responding to night alerts in a strange location was much greater for the responder than the “potential profit” of benefiting a patient-peer in need. It follows that improving members’ sense of personal security during an ERC event may contribute to higher response scores. This need can be answered through real-time communication platforms that support constant contact between ERC responders and the EMS center in the same manner that the EMS center communicates with its own first responders [59-61].

### Limitations

This study has several limitations. The study samples consisted of Israeli and German participants with EAI prescriptions due to a risk of anaphylaxis. Although this represents two distinct cultures and geographies, the generalizability of findings to allergy patients from other geographic areas and cultures is still limited and further studies are needed to this effect.

The sample population included Israeli parents of children with allergy, Israeli adult patients, and German adult patients. Future research including German parents of children with allergy can complete the picture about the barriers and facilitators of participation in ERC events among Israeli and German patients at risk of anaphylaxis.

### Conclusions

An ERC allergy app has the potential to improve outcomes in the case of anaphylactic events, but this depends on patient-peer willingness to respond. The results of this study showed that adults and parents of young patients at risk of anaphylaxis were willing to travel and give their personal EAI to a patient in need when dispatched by an ERC app. The strong positive correlation between shared identity and the willingness to respond for the parent subgroup suggest that an effective ERC allergy app should prefer a local online social community of patients with severe allergy who can respond to ERC alerts, thanks to their proximity and a strong social cohesion, rather than taking national or generally branded approaches. The significant impact of the bystander effect on the willingness to respond on the subgroups and significant lower scores for shared identity reinforce this conclusion.

The decision factors of time and location of emergency event significantly decreased the response score only when interacting with each other. Future examinations of design strategies that would increase ERC members’ sense of personal security may overcome these barriers.

Finally, as proximity-based mHealth interventions take an innovative role in delivering emergency care, identifying its members’ decision factors for participation through patient-centered strategies becomes crucial. This study identified the behavioral and spatial decision factors of severe allergy patients, providing a basis for future research of participation behavior among members of proximity-based mHealth interventions.

## Appendix

Multimedia Appendix 1 Participants’ sociodemographic data.

Multimedia Appendix 2 Shared identity survey.

Multimedia Appendix 3 Anaphylaxis scenarios (four scenarios followed by survey items).

Multimedia Appendix 4 Location familiarity survey.

Multimedia Appendix 5 Validation results of survey scenarios and questionnaire.

## Abbreviations

EAI: epinephrine auto injector
EAR: European Anaphylaxis Registry
EMS: emergency services
ERC: emergency response communities
mHealth: mobile health
SI: shared identity
WR: willingness to respond
