# Supplementary material for: Proximity-Based Emergency Response Communities for Patients With Allergies Who Are at Risk of Anaphylaxis: Clustering Analysis and Scenario-Based Survey Study
Source: JMIR Mhealth Uhealth. 2019 Aug 22;7(8):e13414. doi: 10.2196/13414 (PMC6727626; doi:10.2196/13414)
Supplement: Multimedia Appendix 1 [file mhealth_v7i8e13414_app1.pdf]

Appendix A- Participants' sociodemographic data

People with Allergies Survey

Your Background

Please fill in the following personal background questionnaire

1. How old are you? (in years)
2. Are you: (One- choice question)
    - Male
    - Female
3. Are you: (One- choice question)
    - A person with allergy
    - A parent of allergic children
    - Both parent and a person with allergy

4. Do you or your child allergies require carrying an Epinephrine Auto injector? (One-choice question)
    - Yes
    - No
