# Supplementary material for: Proximity-Based Emergency Response Communities for Patients With Allergies Who Are at Risk of Anaphylaxis: Clustering Analysis and Scenario-Based Survey Study
Source: JMIR Mhealth Uhealth. 2019 Aug 22;7(8):e13414. doi: 10.2196/13414 (PMC6727626; doi:10.2196/13414)
Supplement: Multimedia Appendix 2 [file mhealth_v7i8e13414_app2.pdf]

# Appendix B- Shared Identity Survey

## People with Allergies Survey

### Mobile Emergency Response Communities

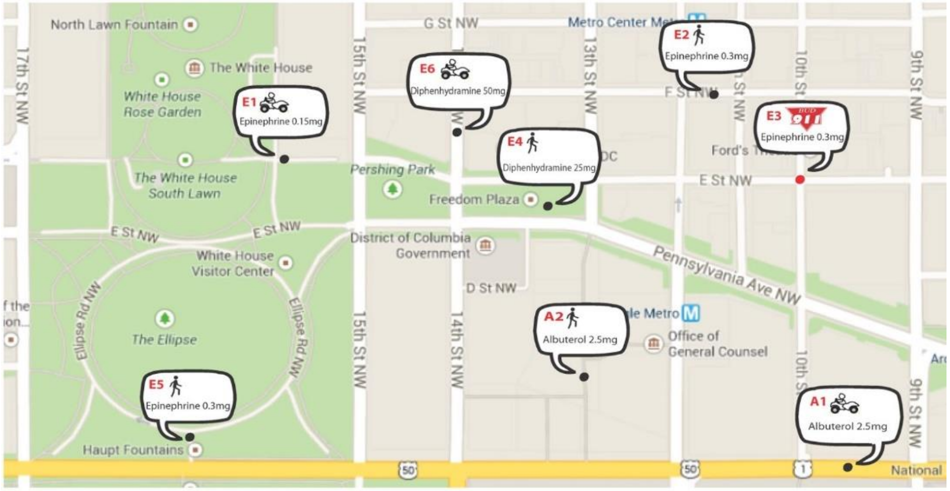


Every person with allergies knows, carrying a personal Epinephrine auto-injector could save lives. Unfortunately, many people with allergies don't carry their injectors at all times, exposing themselves to life threatening situations.

The ERC mobile app initiative at Bar-Ilan University aims to create mobile based communities in which members will give their personal Epinephrine auto-injectors to nearby members who experience severe allergic reactions while not carrying their Auto-injectors. Every treatment delivery between members will be monitored by Emergency Services (MADA) until the rescue workers will arrive at the scene for continuing care.

The members of allergic emergency community will not bare any legal responsibility by participating in the community

## Being a person with allergies

Imagine that you have decided to download the ERC app and become a community member in the Emergency Response Community for people with allergies. As an ERC member, please rank the level of your agreement or disagreement with the following statements.

1. I often think about the fact that me\my child has allergies.

   Strongly disagree — Strongly agree

| | | | | | | | | | |
|---|---|---|---|---|---|---|---|---|---|
| 1 | 2 | 3 | 4 | 5 | 6 | 7 | 8 | 9 | 10 |

2. Being a person\ a parent with allergies is an important part of how I see myself.

Strongly disagree Strongly agree

| | | | | | | | | | |
|---|---|---|---|---|---|---|---|---|---|
| 1 | 2 | 3 | 4 | 5 | 6 | 7 | 8 | 9 | 10 |

3. The fact that I am a person\a parent with allergies is an important part of my identity

Strongly disagree Strongly agree

| | | | | | | | | | |
|---|---|---|---|---|---|---|---|---|---|
| 1 | 2 | 3 | 4 | 5 | 6 | 7 | 8 | 9 | 10 |

4. I feel a bond with other people with allergies.

Strongly disagree Strongly agree

| | | | | | | | | | |
|---|---|---|---|---|---|---|---|---|---|
| 1 | 2 | 3 | 4 | 5 | 6 | 7 | 8 | 9 | 10 |

5. I and the average person with allergies have a lot in common.

Strongly disagree Strongly agree

| | | | | | | | | | |
|---|---|---|---|---|---|---|---|---|---|
| 1 | 2 | 3 | 4 | 5 | 6 | 7 | 8 | 9 | 10 |

6. I feel solidarity with other people with allergies

Strongly disagree Strongly agree

| | | | | | | | | | |
|---|---|---|---|---|---|---|---|---|---|
| 1 | 2 | 3 | 4 | 5 | 6 | 7 | 8 | 9 | 10 |

7. I feel committed to people with allergies

Strongly disagree Strongly agree

| | | | | | | | | | |
|---|---|---|---|---|---|---|---|---|---|
| 1 | 2 | 3 | 4 | 5 | 6 | 7 | 8 | 9 | 10 |

8. . People with allergies have a lot in common with each other.

Strongly disagree Strongly agree

| | | | | | | | | | |
|---|---|---|---|---|---|---|---|---|---|
| 1 | 2 | 3 | 4 | 5 | 6 | 7 | 8 | 9 | 10 |
