# Supplementary material for: Proximity-Based Emergency Response Communities for Patients With Allergies Who Are at Risk of Anaphylaxis: Clustering Analysis and Scenario-Based Survey Study
Source: JMIR Mhealth Uhealth. 2019 Aug 22;7(8):e13414. doi: 10.2196/13414 (PMC6727626; doi:10.2196/13414)
Supplement: Multimedia Appendix 3 [file mhealth_v7i8e13414_app3.pdf]

Appendix C- Anaphylaxis Scenarios (Four scenarios followed by survey items)

Please read the following scenarios carefully before you begin to answer the questions. Notice that some of the questions are worded in the negative and answer accordingly.

Scenario 1- Judith's Emergency Incident (NFM)

Judith worked for a downtown advertising agency in Jerusalem until late. One evening she decided to take a short break in the the central park of Gan Sacher, Jerusalem. Judith was hurrying to her favorite spot, eager for some rest. A few minutes later she noticed an itchy blotch on her left arm which started to swell, followed by a rash that broke out on her face. She realized she is having an allergic reaction to an insect bite. She looked for her Epinephrine auto-injector and realized she had left it at the office. Judith grabbed her smartphone and pushed the ERC app button to request a nearby auto-injector delivery from a community member. Alice, who always carries her Epinephrine auto-injector with her, received Judith's emergency request while jogging on the other side of the park. According to the app's alert message, Alice was not the only community member located next to Judith. A group of nearby community members received the app's emergency request. Alice didn't know if any of them would come to Judith's aid or not.

In light of the app's alert message, if you were Judith:

1. In these circumstances, what is the likelihood that you will continue with your evening Jogging and not respond to Judith's emergency request in Gan Sacher Park?

   Very unlikely — Most likely

1	2	3	4	5	6	7	8	9	10

2. In these circumferences, I would give my personal Auto injector to Judith if she requests for help.

   Very unlikely — Most likely

1	2	3	4	5	6	7	8	9	10

3. In these circumferences, what is the maximum travel time (in minutes) you would invest to give Judith your personal Auto Injector?

Scenario 2- Libi's Emergency Incident (DUS)

During noon, Libi decided to try a new sleeping pill, sold over the counter, to ease her insomnia at home in the distant Neve Yaacov neighborhood, Jerusalem. A couple of minutes after taking the pill, Libi's coughing become so poweful that she started vomiting and realized she was having an allergic reaction to the pill. Although she had been prescribed an Epinephrine Auto-injector, she had never filled the prescription. However, Libi had downloaded the ERC app in case of an allergic reaction, so she pushed the button of the ERC app so a nearby community member would bring her an Auto Injector. Ruth, who kept her Auto injector with her at all times, received Libi's emergency request while she was on a date with her new boyfriend. According to the app's alert message, Ruth was the only community member located next to Libi.

In light of the app's alert message, if you were Ruth:

1. In these circumstances, what is the likelihood that you will continue with your date and not respond to Lib's emergency request in Neve Yaakov Neighborhood?

Very unlikely Most likely

1	2	3	4	5	6	7	8	9	10

2. In these circumstances, I would give my personal auto injector to Libi if she requests for help.

Very unlikely Most likely

1	2	3	4	5	6	7	8	9	10

3.In these circumstances, what is the maximum travel time (in minutes) you would invest to give Libi your personal Auto Injector under these circumstances?

Scenario 3- Adam's Emergency Incident (NFS)

Adam, a 17 year old boy who suffers from acute allergic reactions to almonds, was spending the night at a friend's house in Berlin Mitte. He woke up early and decided to try a new type of cereal he had never seen before. Several minutes after the first bite, Adam felt dizzy. He was having trouble breathing, and his lips began to swell. He realized he was having an allergic reaction, but had left his personal Epinephrine Auto Injector at home. Adam quickly pushed the button of the ERC app that allows members to give their personal auto-injectors to nearby members who experience severe allergic reactions and requested a quick Auto injector delivery from a community member.

Jack, who always carried his personal Auto injector with him, received Adam's emergency request while rushing to a meeting with a promising client. According to the app's alert message, Jack was the only community member within five minutes walking distance to Adam's location.

In light of the app's alert message, if you were Jack:

1. In these circumstances, what is the likelihood that you would not go to this morning meeting and rush to Adam's friend's house in Berlin Mitte?

   Very likely Most likely

1	2	3	4	5	6	7	8	9	10

2. In these circumstances, I would give my personal Auto injector to Adam if he requests for help.

   Very likely Most likely

1	2	3	4	5	6	7	8	9	10

3. In these circumstances, what is the maximum travel time (in minutes) you would invest to give Adam your personal Auto Injector under these circumstances?

Scenario 4- Anne's Emergency Incident (DFM)

Anne worked for a downtown advertising agency. One morning she decided to take a short break in the famous Tiergarten central park. Anne was hurrying to her favorite spot, eager for some rest. A few minutes later she noticed an itchy blotch on her left arm which started to swell, followed by a rash that broke out on her face. She realized she was having an allergic reaction to an insect bite. She looked for her Epinephrine Auto Injector and realized she had left it at the office. Anne grabbed her smartphone and pushed the ERC app button to request a nearby Auto injector delivery from a community member. Alice, who always carries her Epinephrine Auto injector with her, received Anne's emergency request while jogging on the other side of the park. According to the app's alert message, Alice was not the only community member within the reasonable walking time of five minutes from Anne's location in the park; two other community members received the app's emergency request as they were within five minutes from Anne's location. Alice did not know if any of them would come to Anne's aid or not.

In light of the app's alert message, if you were Alice:

1. In these circumstances, what is the likelihood that you will continue jogging in Tiergarten park and not respond to Anne's emergency request?

Very likely Most likely

1	2	3	4	5	6	7	8	9	10

2. In these circumstances, I would not give my personal auto injector to Anne if she requests for help.

Very likely Most likely

1	2	3	4	5	6	7	8	9	10

3. In these circumstances, what is the maximum travel time (in minutes) you would invest to give Anne your personal Auto Injector under these circumstances?
