# Supplementary material for: Proximity-Based Emergency Response Communities for Patients With Allergies Who Are at Risk of Anaphylaxis: Clustering Analysis and Scenario-Based Survey Study
Source: JMIR Mhealth Uhealth. 2019 Aug 22;7(8):e13414. doi: 10.2196/13414 (PMC6727626; doi:10.2196/13414)
Supplement: Multimedia Appendix 4 [file mhealth_v7i8e13414_app4.pdf]

Appendix D- Location familiarity survey (German version)

- Please rate each of the following places with respect to how well you know where these locations are. For example, if the location of a particular place is well known, you would be able to describe accurately how to get to that area, or you could quickly and efficiently guide someone to a specific place in that area.

1. Berlin Neukellen

Completely unfamiliar   Extremely familiar

1	2	3	4	5	6	7	8	9	10

2. Berlin Tiergarten Park

Completely unfamiliar   Extremely familiar

1	2	3	4	5	6	7	8	9	10

3. Berlin Mitte

Completely unfamiliar   Extremely familiar

1	2	3	4	5	6	7	8	9	10

4. Muritz National Park

Completely unfamiliar   Extremely familiar

1	2	3	4	5	6	7	8	9	10

- Using these scales, rate your familiarity according to how easy or difficult it would be for you to recognize a photograph or a picture of each location

1. Berlin Neukellen

Completely unfamiliar   Extremely familiar

1	2	3	4	5	6	7	8	9	10

2. Berlin Tiergarten Park

Completely unfamiliar   Extremely familiar

1	2	3	4	5	6	7	8	9	10

3. Berlin Mitte

Completely unfamiliar Extremely familiar

1	2	3	4	5	6	7	8	9	10

4. Muritz National Park

Completely unfamiliar Extremely familiar

1	2	3	4	5	6	7	8	9	10

- Please rate each cue on the following scales depending on how frequently you see, pass by, or visit the place. A daily or even more frequent encounter would be rated “extremely familiar” while no interaction will yield “completely unfamiliar”

1. Berlin Neukellen

Completely unfamiliar Extremely familiar

1	2	3	4	5	6	7	8	9	10

2. Berlin Tiergarten Park

Completely unfamiliar Extremely familiar

1	2	3	4	5	6	7	8	9	10

3. Berlin Mitte

Completely unfamiliar Extremely familiar

1	2	3	4	5	6	7	8	9	10

4. Muritz National Park

Completely unfamiliar Extremely familiar

1	2	3	4	5	6	7	8	9	10
