# Supplementary material for: Proximity-Based Emergency Response Communities for Patients With Allergies Who Are at Risk of Anaphylaxis: Clustering Analysis and Scenario-Based Survey Study
Source: JMIR Mhealth Uhealth. 2019 Aug 22;7(8):e13414. doi: 10.2196/13414 (PMC6727626; doi:10.2196/13414)
Supplement: Multimedia Appendix 5 [file mhealth_v7i8e13414_app5.pdf]

Appendix E – Validation results of survey scenarios and questionnaire

Survey scenarios and items were validated by a separate sample of 25 patients.

Participants received a short questionnaire which included six multiple choices questions to examine the scenarios' features recognition by the participants. Daytime location of scenarios and number of dispatched ERC responders were identified correctly by 100% of participants. Reliability of the study survey items was measured as follow:

Cronbach's alpha for 8 shared identity, 2 willingness to respond and 3 location familiarity items were .80, .77, .81
